# Monoclonal Antibodies for the Treatment of Ocular Diseases

**DOI:** 10.3390/jcm13195815

**Published:** 2024-09-28

**Authors:** Cristina Henriques, Raquel da Ana, Karolline Krambeck, Sónia Miguel, Antonello Santini, Aleksandra Zielińska, Eliana B. Souto

**Affiliations:** 1 Laboratory of Pharmaceutical Technology, Department of Drug Sciences, Faculty of Pharmacy, University of Porto, 4050-313 Porto, Portugal; 1706241@sal.ipg.pt (C.H.); up202111953@edu.ff.up.pt (R.d.A.); 2 Health Sciences School, Guarda Polytechnic Institute, Rua da Cadeia, 6300-035 Guarda, Portugal; karolline@ipg.pt (K.K.); spmiguel@ipg.pt (S.M.); 3 Department of Pharmacy, University of Napoli Federico II, Via D. Montesano, 49-80131 Napoli, Italy; asantini@unina.it; 4 Department of Biotechnology, Institute of Natural Fibres and Medicinal Plants—National Research Institute, Wojska Polskiego 71b, 60-630 Poznań, Poland; 5 UCD School of Chemical and Bioprocess Engineering, University College Dublin, Belfield, Dublin 4, D04 V1W8 Dublin, Ireland

**Keywords:** monoclonal antibodies, ocular diseases, age-related macular degeneration, diabetic retinopathy, uveitis

## Abstract

Monoclonal antibodies (mAbs) have revolutionized the landscape of cancer therapy, offering unprecedented specificity and diverse mechanisms to combat malignant cells. These biologic agents have emerged as a cornerstone in targeted cancer treatment, binding to specific antigens on cancer cells and exerting their therapeutic effects through various mechanisms, including inhibition of signaling pathways, antibody-dependent cellular cytotoxicity (ADCC), complement-dependent cytotoxicity (CDC), and antibody-dependent cellular phagocytosis (ADCP). The unique ability of mAbs to engage the immune system and directly interfere with cancer cell function has significantly enhanced the therapeutic armamentarium against a broad spectrum of malignancies. mAbs were initially studied in oncology; however, today, treatments have been developed for eye diseases. This review discusses the current applications of mAbs for the treatment of ocular diseases, discussing the specificity and the variety of mechanisms by which these molecules exhibit their therapeutic effects. The benefits, drawbacks, effectiveness, and risks associated with using mAbs in ophthalmology are highlighted, focusing on the most relevant ocular diseases and mAbs currently in use. Technological advances have led to in vitro production methods and recombinant engineering techniques, allowing the development of chimeric, humanized, and fully human mAbs. Nowadays, many humanized mAbs have several applications, e.g., for the treatment of age-related macular disease, diabetic retinopathy, and uveitis, while studies about new applications of mAbs, such as for SARS-CoV-2 infection, are also currently ongoing to seek more efficient and safe approaches to treat this new ocular disease.

## 1. Introduction

**Figure 1:**
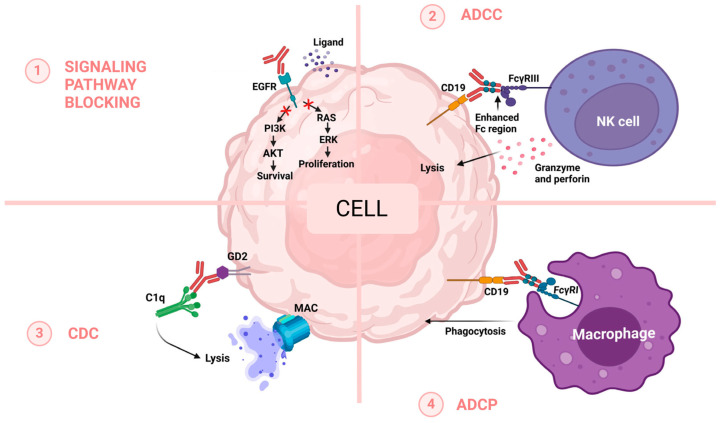
Mechanisms of action for therapeutic monoclonal antibodies (mAbs) in cancer treatment: (1) Inhibition of signaling pathways, targeting key proteins such as AKT (Protein Kinase B) and ERK (Extracellular Signal-Regulated Kinase). (2) Antibody-Dependent Cellular Cytotoxicity (ADCC), where mAbs engage Fc-Gamma Receptors (FcγRIII, FcγRI) on immune effector cells, leading to the destruction of cancer cells. (3) Complement-Dependent Cytotoxicity (CDC), where mAbs activate the complement cascade through C1q, leading to the formation of the Membrane Attack Complex (MAC) and cell lysis. (4) Antibody-Dependent Cellular Phagocytosis (ADCP), where mAbs facilitate the recognition and subsequent phagocytosis of cancer cells by macrophages and other phagocytic cells (modified after Rodríguez-Nava [4]).

Monoclonal antibodies (mAbs) are a class of therapeutic biological products, designed to specifically target and bind certain antigens on the surface of cells, and can be used to treat various diseases by triggering the immune system or regulating cellular processes. The fact that mAbs are monospecific and homogeneous increases the capacity of theranostics compared to polyclonal antibodies, boosting the chances of survival [1,2]. The effector mechanisms encountered in the activity of these types of molecules are illustrated in Figure 1. These therapeutic molecules represent a category of antibodies that consist of a single type of immunoglobulin derived from a solitary B-cell clone and designed to target a particular antigenic site, and were first approved by the Food and Drug Administration (FDA) for commercialization in 1986 [3].

The therapeutic landscape of cancer has been dramatically transformed by the advent of mAbs, which became indispensable tools in the targeted treatment of malignancies. These agents were designed to specifically recognize and bind to antigens expressed on the surface of cancer cells, thereby triggering a cascade of events that can lead to the destruction of these cells. The mechanisms of action (MoA) by which mAbs promote their therapeutic effects are multifaceted and include:

Inhibition of signaling pathways: mAbs can directly inhibit key signaling molecules within the cancer cell, such as protein kinase B (AKT) and extracellular signal-regulated kinase (ERK), thereby disrupting the proliferative and survival signals that are often dysregulated in cancer.

Antibody-dependent cellular cytotoxicity (ADCC): By engaging Fc-Gamma Receptors (FcγR) on immune effector cells, mAbs can recruit these cells to induce the lysis of cancer cells. This process is mediated through the interaction of the Fc portion of the mAb with FcγRIII and FcγRI on natural killer cells and macrophages, respectively.

Complement-dependent cytotoxicity (CDC): mAbs can also activate the complement cascade, starting with complement component 1q (C1q), which binds to the Fc portion of the mAb. This leads to the formation of the membrane attack complex (MAC), which creates pores in the cancer cell membrane, resulting in cell lysis.

Antibody-dependent cellular phagocytosis (ADCP): mAbs facilitate the recognition of cancer cells by phagocytic cells, such as macrophages, through FcγR interactions, leading to the engulfment and destruction of the cancer cells.

These mechanisms highlight the versatility of mAbs in cancer therapy, leveraging both direct and immune-mediated actions to combat malignant disease. The specificity of mAbs to their targets and the variety of mechanisms by which they exert their therapeutic effects underscore their pivotal role in modern cancer treatment. This article will explore these mechanisms in depth, comprehensively understanding how mAbs function as a critical component of cancer treatment strategies.

**Figure 2:**
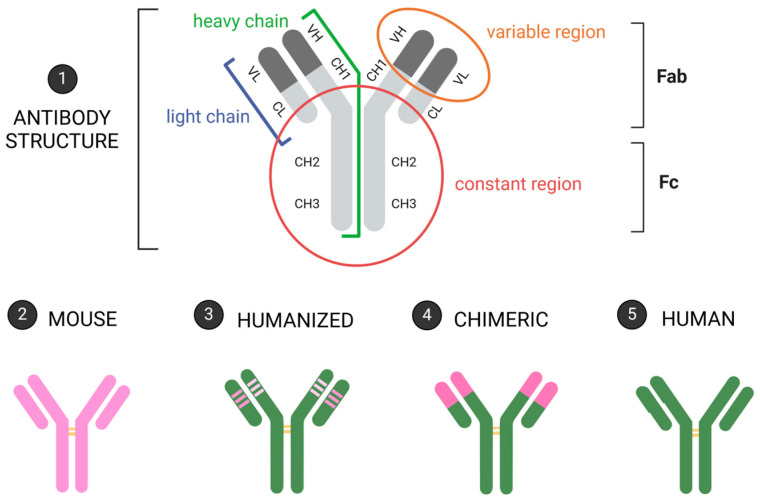
Schematic description of the four main types of mAb compositions for therapeutics: 1—general mAb structure; 2—mouse mAb; 3—humanized mAb; 4—chimeric mAb; 5—human mAb. Pink: mouse sequences; green: human sequences; yellow rectangles: areas of glycosylation [own drawing].

The utilization of hybridoma technology in animals in 1975 marked the introduction of mAb production as a research instrument. Subsequently, in 1986, the murine anti-CD3 mAb was developed for therapeutic purposes. The human immune system’s rejection of these murine mAbs hindered their human application; nevertheless, biotechnology progress has surmounted this challenge, which now enables the synthesis of humanized, chimeric, or fully human mAbs (Figure 2). The developments above resulted in the creation of exceptional therapeutic agents for infectious, rheumatologic, and malignant disorders, which had a significant bearing on the enhancement of human health.

The production of the first mAbs for human use took more than a century to conclude, with numerous experiments being conducted. The critical breakthrough that facilitated further investigations occurred in the 18th century when Dr. Edward Jenner observed that introducing fluid extracted from a smallpox pustule into an individual conferred protection against contracting the disease. In the 1930s and 1940s, with numerous studies by several scientists, the concept of mAbs was created. McMaster and Hudack also isolated agglutin from lymph nodes at that time and discovered that lymphocytes are the principal source of antibody production [5].

In 1970, the first description of mAbs created in the laboratory from murine protein from mice was published, but this type of mAb was not well-tolerated by humans [2]. It was only in 1975 that the first mAb was produced for human use. The first mAb approved for commercialization was muromonab-CD3, a murine mAb used to prevent kidney transplant rejection. The problem with murine mAbs is the risk of immunogenicity because of murine components, limiting their development. To surpass this problem, attention was focused on the development of fully human mAbs. The first fully human mAb was Humira (Adalimumab), accepted for commercialization in 2004 and designed for rheumatoid arthritis treatment [2,6].

The conventional method of producing mAbs is from immunized mice, in which B cells fuse with myeloma cells, thus forming a hybridoma with immortal B cells [6].

With advances in technology, in vitro alternatives have been developed and set up to produce many hybridomas, thus eliminating the use of animals. Although there are various forms of in vitro production, large-scale production of mAbs requires a fiber system. This has many associated problems, such as the activity of mAb production and cell growth. There are currently several strategies to overcome these disadvantages: (i) Developing recombinant antibodies in heterologous expression systems enables the generation and manufacturing of numerous novel antibodies without animal-based stimulation of antigen-specific B cells. In recent years, new recombinant engineering techniques have emerged that make it possible to construct specific mAbs, but with different variations, allowing the development of chimeric, humanized, and fully human mAbs [1]. (ii) Use of phage-based antibodies: This strategy uses the Fab domain of immunoglobins as an antigen-binding site. This Fab domain has variable short and long chains, in which the complementary regions between the chains determine the binding site. The variation in the complementarity sequence allows for the diversity of existing antibodies. The effector capabilities of antibodies are determined by the structure of the Fc domain, which is crucial for their contact with effector cells and the activation of the complement cascade. The differentiation of immunoglobulin isotypes is predicated on the configurations of their Fc domains. Human IgG1, upon binding to cell surfaces, exhibits the capacity to initiate the classical complement cascade. Antibody-dependent cellular cytotoxicity is efficiently stimulated by the identical isotype, a process facilitated by leukocytes that harbor the requisite Fc receptors. This mechanism plays a crucial role in the destruction of target cells that are attached to the monoclonal antibody [1].

Therapeutic mAbs have only recently been implemented in ophthalmology to treat angiogenic and inflammatory diseases [7]. The recent understanding of the molecular biology of numerous ophthalmic diseases further supports the concept of mAb application in ophthalmology.

The cytokine tumor necrosis factor-alpha (TNF-a), or TNF-A, is implicated in the pathogenesis of autoimmune ocular inflammatory diseases, including uveitis, in the context of inflammatory ocular conditions. To treat uveitis and other inflammatory eye diseases, the chimeric anti-TNF-a mAb infliximab and the more recent adalimumab are effective. In the context of ocular angiogenesis, the cytokine vascular endothelial growth factor (VEGF) is a significant contributor to the formation of new blood vessels in several neovascular disorders, such as diabetic retinopathy (DR) and age-related macular degeneration (AMD).

Vascular endothelial growth factor (VEGF), referred to as vascular permeability factor (VPF), was initially characterized as a mitogen unique to endothelial cells. VEGF is synthesized by various cell types, including tumor cells, macrophages, platelets, keratinocytes, and renal mesangial cells [8]. Its roles extend beyond the vascular system, i.e., being involved in normal physiological processes, e.g., bone formation, hematopoiesis, wound healing, and development. The evidence that VEGF has a significant role in tumor angiogenesis has led to the development of many anti-VEGF treatments aimed at inhibiting tumor growth and angiogenesis. The impact of VEGF on retinal disorders is significant. It has been associated with numerous retinal diseases and conditions, including prevalent disorders such as AMD and DR, and less common ailments such as retinopathy of prematurity, sickle cell retinopathy, and retinal vascular occlusion, as well as being a non-causal yet significant secondary factor in neovascular glaucoma and inherited retinal dystrophies. These disorders, each possessing essential angiogenic elements, collectively contribute to the predominant cause of irreversible vision loss [9]. In experimental and clinical studies, it has been reported that the full-length mAbs, anti-VEGF bevacizumab and VEGF-fragment ranibizumab, promote significant regression of intraocular neovascularization.

This review discusses the benefits, drawbacks, effectiveness, and risks associated with using mAbs in ophthalmology, focusing on the most relevant ocular diseases described below.

## 2. Ocular Diseases

### 2.1. Age-Related Macular Degeneration

Age-related macular degeneration (AMD) is a chronic and progressive condition affecting the central retina and is a leading cause of global vision impairment. It is the predominant cause of disability among adults aged 55 and above in developed nations. The disease does not show symptoms during the initial phases and gradually advances. Most of the vision impairment occurs in the later stages. The main risk factor for AMD is age [10].

AMD is the most prevalent cause of vision loss and is an age-related condition. However, people can develop macular degeneration at younger ages because of numerous factors. Its manifestations and pathogenesis are diverse, so the term used is just a way of encompassing this diversification in a single disease. Despite numerous studies carried out over time, the information obtained about macular degeneration has not yet been fully answered. Its pathogenic mechanisms are still being discovered, but it is known that there are several factors, such as oxidative stress, diet, smoking, and the extracellular matrix, among others, which are possible mechanisms for some types of macular degeneration [11].

Several instruments employed in genetics and oncology have yielded notable advancements in enhancing the prospects of therapies that can effectively mitigate macular degeneration in a substantial proportion of the presently vulnerable population. In addition, rarer Mendelian conditions with clinical and histopathological overlap with macular degeneration allow for a better understanding of the progression of the disease [12,13].

This disease is particularly prevalent since the population’s living conditions have improved. As it is a disease that appears at older ages, the longer people live, the greater the number of people affected. Multiple epidemiological studies indicate that the prevalence of macular degeneration is less than 5% among those below the age of 65. Nevertheless, the frequency of its occurrence significantly rises in older persons, affecting more than 35% of people over 75 to varying extents. Over 10% of individuals aged 80 and beyond will experience significant visual impairment during the advanced phases of AMD [12,13].

In the context of AMD, the disease is typically categorized into two main stages: early AMD and late AMD. These stages are defined based on the presence and severity of certain clinical features observed during an eye examination. Early AMD is characterized by the presence of drusen (yellow deposits under the retina) and pigment changes in the retina. At this stage, there may be no noticeable vision loss or only minimal changes in central vision. Late AMD is further divided into two forms. (i) Wet (neovascular) AMD: This form is characterized by the growth of abnormal blood vessels under the retina. These vessels can leak blood and fluid, lifting the retina from its normal position and causing damage, leading to a rapid loss of central vision. (ii) Dry (geographic atrophy) AMD: This form involves a gradual breakdown of cells in the retina, resulting in a slow loss of central vision. In dry AMD, the retinal cells die, leaving a blank spot in the central vision, which worsens over time [14].

When referring to the initial or late stages of AMD in the document, it is important to clarify that the initial stage typically refers to early AMD, with early signs of the disease but often without significant vision loss, while the late stage refers to either wet or dry AMD, where there is active damage to the retina and noticeable vision loss, particularly in central vision, which is critical for tasks such as reading and recognizing faces. Understanding these stages is crucial for both clinicians and patients, as it helps in planning appropriate interventions and managing expectations regarding the potential outcomes of treatment [15,16].

AMD is typically identified by the existence of widespread drusen, frequently accompanied by pigmentary abnormalities. Drusen are pale yellow sub-retinal pigment epithelial (RPE) deposits of lipids and proteins. They can differ in size and location and can be extramacular or central. Recent studies suggest that an increase in the volume of central drusen leads to an increased risk of AMD progression, which is why they are considered biomarkers of AMD [10,17]. They are commonly observed in older individuals [15].

Two classification schemes have been devised to evaluate the likelihood of disease development from the initial to the more advanced phases: an intricate plan that typically requires meticulous examination of photographs for implementation and a streamlined system that is straightforward to apply in a clinical setting. Both schemes relate the risk of advanced stages to the drusen’s size and pigmentary changes. In the Rotterdam system, there are nine levels of severity, as described in the Age-Related Eye Disease Study (AREDS). Meanwhile, in the Ferris scheme, there is an increase in the risk of progression every 5 years in patients over 55 [18].

The etiology of the disease remains uncertain; however, numerous hypotheses have been advanced, encompassing oxidative stress, mitochondrial dysfunction, and inflammatory mechanisms [15].

Multiple modalities exist for the treatment. Recent population data have demonstrated a 50% reduction in legal blindness caused by age-related macular degeneration in certain nations following the advent of drugs that decrease vascular endothelial growth factor (VEGF), known as VEGF antagonists. Currently, intravitreal anti-vascular endothelial growth factor drugs are considered the first-line therapeutic strategy for treating eye diseases such as AMD. AMD requires treatment in order to reverse or minimize visual loss and stabilize or improve visual function [19].

In 2006, pivotal clinical trials showed that monthly intravitreal ranibizumab injections effectively mitigate vision loss in over 95% of patients and substantially enhance vision in 40% [20]. Bevacizumab, initially created for treating colon cancer, is closely linked to the original ranibizumab molecule. It is currently commonly used off-label as a more affordable alternative to ranibizumab, as it has shown comparable effectiveness. Furthermore, significant progress has been achieved in comprehending the epidemiology, risk factors, and genetics of age-related macular degeneration alongside its treatment [16].

### 2.2. Diabetic Retinopathy

Diabetic retinopathy (DR) is one of the most significant complications of diabetes mellitus and is one of the leading causes of visual loss in people of working age. It is related to the degree and duration of diabetes and is diagnosed through the appearance of abnormalities in the blood vessels present in the retina.

DR is categorized into two stages. Non-proliferative diabetic retinopathy (NPDR) is the early stage of DR, characterized by heightened vascular permeability and blockage of capillaries in the retina. At this point, fundus photography can detect retinal abnormalities such as microaneurysms, hemorrhages, and hard exudates, even if individuals do not show any symptoms. Proliferative diabetic retinopathy (PDR) is the final and most critical stage of diabetic retinopathy, which occurs when new blood vessels form in the eye due to diabetes. This can lead to severe vision problems when these aberrant veins bleed into the vitreous (vitreous hemorrhage) or cause the retina to detach due to pulling (tractional retinal detachment).

Diabetic macular edema (DME) is the primary reason for vision loss in patients with diabetic retinopathy (DR). DME is characterized by fluid accumulation in the macula, swelling, or thickening. This can happen at any stage of DR and leads to distorted visual images and reduced visual acuity [21].

**Figure 3:**
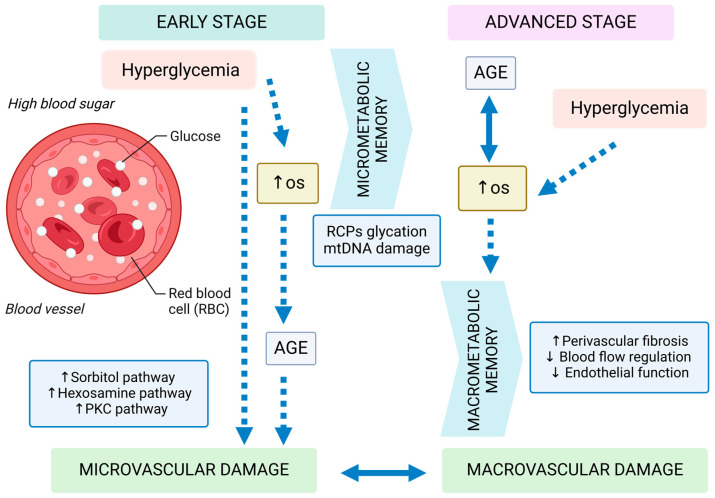
A comprehensive depiction of the various causal factors of hyperglycemia, AGEs, and oxidative stress (OS) across the various stages of diabetes mellitus. A “micro” metabolic memory, which operates at the mitochondrial level, is thought to liberate the effects of AGEs from the constraints of chronic hyperglycemia. Conversely, a “macro” metabolic memory emerges from the prolonged interplay between oxidative stress and glycation at the tissue and vessel levels, serving as a link between damage to both large and small blood vessels. AGEs stands for advanced glycation end products, mtDNA for mitochondrial DNA, OS for oxidative stress, PKC for protein kinase C, and RCPs for respiratory chain proteins; ↑, increase, ↓ decrease [own drawing].

Multiple biochemical routes have been suggested to establish the connection between hyperglycemia and related microvascular problems (Figure 3). The factors involved in this process are polyol buildup, the creation of advanced glycation end products (AGEs), oxidative stress, and the activation of protein kinase C (PKC). It is hypothesized that these processes influence disease progression by affecting cell metabolism, signaling, and growth factors [22].

In addition to the loss of vision, DR is associated with various economic and social consequences, as a person with DR is more likely to be unemployed or unable to find a job. In addition, the costs associated with a person with diabetes and blindness are pretty high, estimated at USD 500 million in the 1990s [23].

Currently, there are several strategies for treating DR to control microvascular complications, such as intravitreal pharmacological agents, laser photocoagulation, and vitreoretinal surgery. Despite these strategies, intravitreal administration of anti-VEGF agents is the mainstay of DR therapy at all stages, as it allows visual improvement with fewer ocular side effects than laser therapy. However, only 29 percent of patients showed significant improvements when treated with anti-VEGF due to the various possible biochemical pathways [21].

Prevention of DR is also a widely used approach in patients with diabetes. In this study of 1441 patients with type 1 diabetes, patients were randomized to receive intensive or conventional therapy for an average of 6.5 years. There was a notable reduction in the rate of development or progression of retinopathy among patients assigned to intensive treatment, and also a reduction in the progression of diabetic nephropathy and neuropathy [24].

### 2.3. Uveitis

Uveitis is characterized by inflammation of the uveal tract that affects the retina, ciliary body, iris, and choroid. Patients with uveitis experience various symptoms, including blurred vision, floaters, redness of the eye, and spontaneous and light-evoked pain [25,26]. This is a frequently occurring eye condition characterized by inflammation, with documented yearly rates ranging from 17 to 22.6 cases per 100,000 people.

Uveitis can be classified according to its laterality, clinical course, and location; the correct diagnosis requires considering all three of these characteristics and the patient’s symptoms [27].

Concerning the location, uveitis can be classified as (i) ‘anterior’ to iridocyclitis, denoting the condition affecting the iris or ciliary body; (ii) ‘posterior’ to choroiditis or retinochoroiditis when affecting the choroid or, by extension, the retina (retinal vasculitis may or may not develop from posterior uveitis); (iii) “intermediate” uveitis refers to an inflammation that is confined to the pars plana, ciliary body, vitreous, or peripheral retina; and (iv) “panuveitis” when denoting an infection that affects two or more of these segments.

Uveitis is categorized into three types based on its clinical course: (i) “acute,” denoting less than three months; (ii) “chronic,” signifying a duration exceeding three months; and (iii) “recurrent,” signifying the recurrence of an acute exacerbation after the full resolution of a preceding episode.

Regarding laterality, the disease can manifest as either (i) “unilateral,” denoting simultaneous involvement of both eyes, or (ii) “bilateral,” signifying the potential for recurrences in the contralateral eye.

Genetic, geographical, and environmental factors significantly impact the types, clinical associations, and etiology of uveitis within a specific population. Consequently, research on the distribution of uveitis types and causes is crucial to assist physicians in adopting a targeted and suitable approach to diagnosis, treatment, and investigation [27]. Some of the best-known examples include: (i) onchocerciasis, a nematode-borne infection transmitted by insects and one of the leading causes of uveitis in specific regions of Africa, Central and South America, and Yemen, is geographically distributed; (ii) Japan and the Mediterranean basin have a comparatively high incidence of Behcet’s disease; and (iii) specific types of uveitis, including acute anterior uveitis and HLA-B27, are genetically associated with particular HLA antigens. This disease has been significantly studied recently, and some of these studies have compared their results with those from other regions. Despite this, a global comparison does not yet exist [28].

In uveitis, TNF-α is a crucial factor. The rat model of endotoxin-induced uveitis (EIU) exhibits a discernible early increase in TNF-α levels in both aqueous humor and serum [29]. Furthermore, acute uveitis is observed in mice and rats injected intravitreally with TNF-α after polymorphonuclear granulocyte infiltration. TNF-α was quantified in uveitis patients’ aqueous humor and serum [30]. Despite its insignificance in aqueous humor, the authors deduced that TNF-α is elevated in the serum; this immunologic pattern appears to be linked to chronic uveitis recurrences. Based on this fact, inhibiting TNF-α seems to be a potentially effective therapeutic strategy for uveitis.

### 2.4. COVID-19

Ocular manifestations may also result from COVID-19 infection and were reported to be associated with a dysregulated immune response to the initial infection, increased propensity for a hypercoagulable state leading to prothrombotic events, corticosteroid-induced immunosuppression, or as a result of comorbidities [31]. Several ophthalmic diseases, such as conjunctivitis, scleritis, uveitis, endogenous endophthalmitis, corneal graft rejection, retinal artery and vein occlusion, non-arteritic ischemic optic neuropathy, glaucoma, and neurological and orbital sequelae, were reported secondary to SARS-CoV-2. The virus can be detected by polymerase chain reaction (PCR) in several ocular structures (e.g., aqueous and vitreous humors, tears, retina, and the optic nerve) and, besides glucocorticoids, antiviruses, and vaccines, molecules such as interleukin-6, Janus kinase inhibitors, and mAbs have been proposed as treatment options [32]. Examples of the successful use of mAbs in clinical trials include bamlanivimab, casirivimab, etesevimab, imdevimab, and sotrovimab [33].

## 3. Monoclonal Antibodies and Their Application in Ocular Diseases

### 3.1. Bevacizumab

Bevacizumab is an approved VEGF inhibitor for the treatment of malignancy. Off-label, it is widely utilized as an intravitreal injection to treat eye diseases due to its cost-effectiveness. It is a recombinant monoclonal antibody with human structural regions and the binding regions of a murine antibody to the active isoforms of VEGF, mainly the VEGF-A isoform, inhibiting angiogenesis and preventing tumor growth and proliferation. It is produced through recombinant biotechnology using Chinese hamster ovary cell lines, thus maintaining the affinity of the parent antibody while increasing the biological half-life [34].

The mechanism of action of bevacizumab is based on its binding to circulating VEGF to prevent its receptors, such as VEGFRs, from binding. Thus, the formation of new blood vessels is prevented, and the blood supply to tumor tissues is limited. As well as limiting the blood supply, it decreases tissue interstitial pressure, increases vascular permeability and the administration of chemotherapeutic agents, and favors the apoptosis of tumor endothelial cells [34,35].

Recent studies have reported advantages in the use of bevacizumab for AMD [36,37,38]. In clinical settings, bevacizumab is administered intravenously in doses between 5 and 15 mg/kg to treat tumors. In addition to treating tumors, researchers are studying the possibility of using it in patients with AMD and diffuse diabetic macular edema at a dose of 1.25 mg [35].

Gumus et al. (2022) [39] evaluated the early effects of intravitreal bevacizumab (IVB) injection on intraocular pressure (IOP), central corneal thickness (CCT), corneal volume (CV), anterior chamber depth (ACD), and iridocorneal angle (ICA). The study incorporated forty-two eyes from a total of forty-two patients, with the patients’ mean age being 60.1 ± 7.4 years. The average intraocular pressure (IOP) readings at 1 h and day one before and following injection were 15.4 ± 2.4 and 14.7 ± 2.3, respectively. Significant differences were observed in the mean IOP, CCT, and CV values one hour after injection compared to pre-injection values (*p* < 0.05, *p* < 0.05, and *p* = 0.02, respectively). In contrast, the average ACD and ICA values were found to be substantially reduced compared to their pre-injection values one hour later (*p* = 0.01 for both). No statistically significant variations were observed across all parameters on the initial day following injection. One hour after the injection of bevacizumab (2.5 mg/0.1 mL), transient increases in IOP and decreases in ACD and ICA were observed. Transient increases in CCT and CV may be associated with an increase in IOP. The alterations revert to their initial values on the initial day following the injection.

### 3.2. Ranibizumab

Ranibizumab is a recombinant humanized mAb produced in an *Escherichia coli* expression system with specific binding to the active forms of VEGF-A, thus preventing interactions with endothelial cell receptors and preventing the formation of blood vessels [40].

Ranibizumab is used in the treatment of ocular pathologies because, as it consists of a humanized antibody fragment, its penetration into the retina of the eye is more effective, which allows for greater efficacy in the treatment of diseases such as AMD, central retinal vein occlusion (CRVO), central retinal vein branch occlusion (CRVO), and DR [41]. Ranibizumab allows for an improvement in visual acuity and a slight regression of retinal changes, with the recommended type of treatment ranging from 0.3–0.5 mg per dose for four weeks through intravitreal injections.

Its safety and efficacy profiles are often compared with those of bevacizumab. Although both mAbs have the same efficacy in the treatment of eye diseases and bevacizumab is more affordable, it is not authorized for use in the treatment of eye diseases, so ranibizumab is the most widely used due to its well-established safety and efficacy profile through rigorous clinical trials [42].

Ranibizumab is the first anti-VEGF medication authorized for the treatment of diabetic retinopathy with or without DME by the FDA [43]. Although bevacizumab was approved earlier than ranibizumab, it was not designed for intraocular use. Ranibizumab therapy improves retinal function and is an effective therapeutic agent for treating DME. In contrast, recent trials have shown that IVT injections can substantially induce the resolution of edema and enhance the VA of patients with DME.

### 3.3. Adalimumab

Adalimumab is an antibody derived from humans that is manufactured using recombinant deoxyribonucleic acid (DNA) technology. By inhibiting TNF-activity, adalimumab, a human mAb, is a biological therapy to reduce symptoms and delay the onset of structural damage in patients with moderate to severe rheumatoid arthritis. It is one of three TNF-α blocking agents, with adalimumab being the most recently developed.

Adalimumab exhibits a strong affinity for human TNF-α, an endogenous cytokine that plays a role in the acute stage of inflammatory immune reactions. Adalimumab inhibits TNF-alpha via its particular affinity by interacting with the p55 and p75 cell-surface TNF receptors. Adalimumab is also capable of lysing TNF-expressing cell surfaces in vitro in the presence of a complement.

TNF-α seems to be predominantly involved in pathological inflammation and tissue injury. TNF-α is widely distributed in all actively inflamed tissues, including the synovial fluid of individuals with rheumatoid arthritis or plaquegiria, as well as the eye in cases of acute uveitis.

On the contrary, adalimumab exhibits a particular anti-TNF-α effect. It does not impede the production of the lymphotoxin TNF-β by lymphocytes, which impacts a diverse array of cells (although TNF-β does regulate numerous biological responses in response to TNF-α stimulation). These biological responses, in particular, profoundly affect three adhesion molecules that are accountable for leukocyte migration: intercellular adhesion molecule, vascular cell adhesion molecule (VCAM-1), and endothelial leukocyte adhesion molecule (ELAM-1).

The FDA approved adalimumab in 2002 to mitigate symptoms and impede the advancement of structural harm among individuals diagnosed with moderate to severe rheumatoid arthritis. Still, in 2004, a new approval was requested for the treatment of psoriatic arthritis, and an application was also made to the EMA for its commercialization on European soil.

In addition to its aforementioned therapeutic indications, adalimumab has therapeutic possibilities in treating psoriasis, ankylosing spondylitis, and inflammatory bowel diseases [44].

The impact of adalimumab on the frequency of anterior uveitis flare-ups in patients with active ankylosing spondylitis (AS) was assessed [45]. The authors analyzed the medical records of 1250 patients with active AS who were participating in an international, uncontrolled, open-label clinical trial of adalimumab (40 mg every other week for a maximum of 20 weeks) and had a history of anterior uveitis as diagnosed by ophthalmologists. Their findings indicate that adalimumab significantly prevented anterior uveitis flares in patients actively experiencing AS. However, due to the noncomparative nature of the study, it is not feasible to determine whether adalimumab exhibits a superior safety profile or is exceptional compared to other biologics.

The transition from one anti-TNF agent to another is a second prevalent subject in biologics. In a recent publication, Takase et al. [46] detailed the successful transition of Behcet patients from infliximab to adalimumab. The authors examined patients who received infliximab treatment, specifically those with cyclosporine-resistant ocular lesions, and maintained clinical remission. All subjects under consideration maintained optimal control until the patients encountered recurrent infusion reactions related to infliximab. In such situations, the cessation of treatment immediately precipitated additional ocular attacks. Once more, clinical remission was observed after the treatment was changed to adalimumab, indicating that this medication may serve as a secure and productive substitute for infliximab in hypersensitive patients. While the findings regarding Behcet disease exhibit potential, the absence of a controlled trial and the impossibility of comparing the properties of various anti-TNF-α agents restrict their applicability to refractory cases of the disease.

An analogous hypothesis regarding refractory uveitis can be put forth. Diaz-Llopis et al. (2008) [47] evaluated the safety and efficacy of adalimumab in treating refractory autoimmune uveitis in a pilot study. For one year, nineteen patients were enrolled and a 40 mg subcutaneous adalimumab injection was administered every other week. All patients had successfully decreased the dosage of the concurrent immunosuppressive medications by a minimum of 50%. However, severe adverse events were not reported in any patient as a result; only local minor side effects were observed at the site of subcutaneous injection.

### 3.4. Infliximab

The chimeric monoclonal antibody (mAb) infliximab comprises a human IgG1 constant region and a murine antigen-binding region; it binds specifically to human TNF-α [48].

Infliximab can bind to two molecules of TNF-α, which can be membrane-bound or soluble, producing a stable bond between the two compounds. One molecule of TNF-α can have up to three molecules of Infliximab associated with it, and when this happens, TNF-α cannot bind to its receptors, preventing biological activity [48].

It is suspected that, due to the action of the IgG1 in the infliximab molecule, the binding between the two compounds causes lysis of TNF-α-producing cells through the activation of complement-dependent or antibody-dependent cell-mediated cytotoxicity. In addition, infliximab promotes bonds with activated peripheral blood lymphocytes and T cells, preventing their apoptosis [48].

Miraldi Utz et al. (2019) [49] evaluated the efficacy of long-term infliximab treatment for refractory non-infectious pediatric uveitis and the influence of treatment adherence on disease control.

Infliximab, a chimeric human/murine monoclonal antibody to TNF-α, inhibits binding to TNF receptors [50] by binding to free and membrane-bound protein variants and instigating a conformational change. Infliximab has been employed for numerous years in treating pediatric uveitis, yielding reported response rates ranging from 0% to 100% [51,52]. To effectively manage and treat uveitis, it is often necessary to adhere to complex medication regimens and attend regular appointments. Ensuring compliance with treatment regimens and subsequent evaluations is critical for successfully managing uveitis [53,54]. Children constitute a susceptible demographic, and treatment efficacy may be significantly contingent upon the collaboration between caregivers and clinicians to foster adherence. The potential for uveitis, with or without juvenile idiopathic arthritis, to result in permanent and residual disability has lifelong repercussions for these children. A clinician can monitor infusion administration, which is one of the benefits of Infliximab. Therefore, it is crucial to comprehend the effect that adherence has on the efficacy of treatments and the management of diseases. A single-center investigation of children with recalcitrant pediatric non-infectious uveitis is utilized to assess the long-term effectiveness of Infliximab in maintaining inflammatory control and the effect on treatment adherence. The patient population included in this study is more significant than that reported in the pertinent literature [49].

The effectiveness of infliximab in treating Behcet’s disease and other intraocular inflammations has been demonstrated in numerous studies. Mushtaq et al. (2007) [55] presented the initial clinical prognosis of three Behcet’s disease patients previously treated with infliximab but subsequently transferred to adalimumab. All patients maintained stable visual acuities and were free of recurrences following the administration of adalimumab. The authors hypothesize that adalimumab may assist in the maintenance and remission of Behcet’s disease.

### 3.5. Certolizumab

Certolizumab pegol is a humanized recombinant monoclonal antibody. The Food and Drug Administration (FDA) of the United States has approved its use in treating Crohn’s disease, rheumatoid arthritis, ankylosing spondylitis, and psoriatic arthritis. Currently, the available data regarding the safety and effectiveness of certolizumab pegol in managing ocular inflammatory diseases are notably scarce [56]. Certolizumab pegol (CZP) is a mAb formed by attaching a Fab fragment of a humanized murine monoclonal antibody to two molecules of PEG, a non-immunogenic and non-toxic polymer. CZP specifically recognizes and neutralizes human TNF, both in soluble and membrane-bound form, in a dose-dependent manner. The Fab region does not induce antibody-mediated cytotoxicity, and its structure allows CZP to have a plasma half-life of approximately 14 days, thus increasing its distribution in smooth tissues [57].

Certolizumab is effective in reducing the migration of leukocytes to inflammatory sites, as well as modulating the expression of adhesion molecules and the production of pro-inflammatory cytokines. These effects attenuate the inflammatory response in conditions such as rheumatoid arthritis, Crohn’s disease, and ankylosing spondylitis, for which certolizumab is indicated [58].

Pegol certolizumab is an innovative TNF antagonist, an antigen-binding fragment of a humanized monoclonal antibody, which is recombinant, polyethylene glycosylated, and neutralizes TNFα. In essence, certolizumab differs from other TNF antagonists (adalimumab and infliximab) because it is not a complete antibody and lacks a potentially immunogenic Fc segment.

The clinical case of a 64-year-old female patient was reported. The patient had bilateral decreased visual acuity (VA), with the majority of the impairment occurring in her left eye [59]. The patient’s medical history was notable for the presence of seropositive rheumatoid arthritis (RA), which had been effectively managed with methotrexate and certolizumab for the preceding three years. There was no prior ocular history of the patient. In addition to topical steroid injections, the patient was treated with anti-glaucoma drugs. A resolution was reached for the anterior uveitis, and the intraocular pressure (IOP) was normalized. The certolizumab was discontinued in collaboration with the rheumatologist, while the methotrexate dosage was increased. The topical steroidal treatment was discontinued after a tapering process. Two months after the cessation of certolizumab treatment, both eyes exhibited reduced uveitis and improved visual acuity to 20/32. However, the patient’s macular edema continued to worsen despite periocular injections of 40 mg triamcinolone acetonide in both eyes; the macular edema was only partially resolved.

This molecule was also tested in a trial of patients with refractory non-infectious uveitis [60]. Even though the patients presented with a variety of uveitis types and underlying causes, they were all resistant to or intolerant of several treatments. Certolizumab was administered to all patients as a treatment option following the ineffectiveness of prior agents, which included at least one TNF inhibitor. Other treatments were discontinued due to the following: therapy intolerance, drug infusion-induced allergic reaction, adverse event development (including skin rash, nausea, headache, fatigue, and shortness of breath), insufficient inflammatory control, and reactivation of the patient’s systemic diseases. Adalimumab, the only TNF inhibitor approved by the FDA, was ineffective in treating non-infectious uveitis in all cases.

### 3.6. Bamlanivimab

Bamlanivimab is a specific monoclonal antibody against the spike protein (S protein) of severe acute respiratory syndrome-coronavirus-2 (SARS-CoV-2). This mAb was derived from the convalescent plasma of COVID-19 patients [61].

This antibody is neutralizing and is one of the most potent antibodies approved by the FDA. It is also combined with another antibody, etesivimab, to fight the SARS-CoV-2 virus [62].

### 3.7. Casirivimab

Casirivimab is a recombinant mAb that neutralizes human immunoglobulin G1 to the spike protein of the SARS-CoV-2 virus. This antibody binds to the S1 subunit of the spike protein-binding region, blocking the virus from binding to its receptor, thus preventing the virus from replicating [63]. Despite its importance in the fight against SARS-CoV-2, the risk of severe side effects has meant that the isolated use of Casirivimab has not been authorized. To be permitted for use, Casirivimab must be combined with another mAb, imdevimab, at 600 mg + 600 mg. This combination is used as a single intravenous infusion.

The two combined mAbs easily bind to different epitopes in the spike protein-binding region, with each antibody having the ability to block the protein from binding to the virus almost entirely [63].

### 3.8. Imdevimab

Imdevimab, a monoclonal antibody (mAb) targeting the spike (S) protein of the causative agent of coronavirus disease 2019 (COVID-19), severe acute respiratory syndrome coronavirus 2 (SARS-CoV-2), was among the mAbs suggested for the clinical application of COVID-19 throughout the pandemic. On 21 November 2020, it was proposed to use a mixture of Imdevimab and another mAb, casirivimab, to combat COVID-19 cases. This mixture, when administered, has been shown to decrease viral load and reduce the risk of hospitalization. In addition to these effects, it also prevents virus-induced pathological sequelae [64,65].

### 3.9. Brolucizumab

Brolucizumab is used in the treatment of AMD and is an anti-vascular endothelial growth factor (VEGF) and single-chain antibody fragment of approximately 26 kDa [66]. In recent clinical phase 3 studies, brolucizumab and aflibercept were compared for 96 weeks, with both brolucizumab and aflibercept improving vision and providing efficient disease control, demonstrating a well-tolerated safety profile [67]. Based on its efficacy and safety data, brolucizumab was approved in 2019 in the United States and has now been approved in more than 40 countries, including the European Union [66].

Brolucizumab is a powerful drug; however, ocular inflammation is a severe side effect of this drug. In a clinical study, Hawk, which compared brolucizumab and aflibercept, there were more cases of serious adverse events such as intraocular inflammation, retinal detachment, endophthalmitis, retinal artery thrombosis, and retinal artery occlusion in the study group that used brolucizumab [68].

Another study reported that brolucizumab was administered for 9 months, with 6 mg of brolucizumab injected every 8 to 12 weeks. It was found that there were several adverse events, including 2.6% of cases with intraocular inflammation, one case of retinal detachment, and one of retinal artery occlusion [68]. It has also been suggested that the frequency of doses administered is related to the observed adverse events.

Several studies show that brolucizumab demonstrates good efficacy in the treatment of diabetic retinopathy [69,70,71,72]. The KINGFISHER randomized clinical trial, in which 517 participants were treated for 52 weeks with 6 mg of bolucizumab or 2 mg of aflibercept every 4 weeks, showed better results with brolucizumab and did not identify any extra concerns about the safety of this drug [73].

### 3.10. Faricimab

Faricimab was the first bispecific antibody introduced in 2022 for the treatment of eye diseases such as AMD [18]. Faricimab selectively binds to VEGF-A and angiopoietin-2 (Ang-2), thus influencing two different receptor routes. By neutralizing Ang-2, the stability of blood vessels is restored, which leads to a decrease in permeability and neovascularization [74].

Several studies, including phase 3 clinical trials (YOSEMITE/RHINE, TENAYA, and LUCERNE), have reported an advantage in the use of faricimab, suggesting good efficacy and safety in the treatment of ADM and diabetic macular edema. This suggests that faricimab’s dual mechanism of action can bring many advantages to treatment [75,76,77,78,79].

### 3.11. Aflibercept

Aflibercept, a human protein with a molecular weight of 115 kDa, binds to circulating VEGF, “trapping” the molecule and suppressing the activity of VEGF-A and VEGF-B, as well as the placental growth factor. This inhibits the growth of new blood vessels. It was approved for the treatment of eye diseases in 2012. Aflibercept is now used for neovascular glaucoma, diabetic macular edema, myopic choroidal neovascularization, retinal vein occlusion, and even for retinopathy for prematurity [80].

The effectiveness of aflibercept has been demonstrated in numerous studies. DeCross et al. evaluated the efficacy and durability of aflibercept over 2 years in a study for the treatment of neovascular AMD. They concluded that there was an improvement in vision, even in the first year of treatment, demonstrating that this therapy can be a strategy in the treatment of ADM with satisfactory results [81].

In a recent study, an intravitreal dose of 8 mg of Aflibercept was used for 96 weeks in a phase 3 clinical trial (PULSAR) in the treatment of AMD, demonstrating safety and efficacy at the proposed extended dosing intervals [82].

Administration is usually intravitreal in different rounds (2 mg per injection), separated monthly during the first three months and bi-monthly during the rest of the treatment [83]. No cytotoxic effects have been observed in in vitro studies on corneal endothelial cells [84].

Although adverse side effects have been observed from its administration, among which we can highlight eye irritation, eye hemorrhages, vitreous detachment, blurred vision, and local swelling, some more serious side effects can also occur, such as increased eye pressure, retinal detachment, thromboembolic events, and traumatic cataracts, although some of these can be attributed to the administration procedure by injection. However, complications are rare at less than 0.1% [85].

### 3.12. Etanercept

Etanercept, a recombinant fusion protein, neutralizes TNF-α before it binds to its receptor by fusing the Fc domain of human IgG1 with extracellular human p75 TNF receptors; infliximab, a chimeric IgG1 monoclonal antibody to TNF-α from mice to humans, neutralizes both the soluble and membrane-bound forms of TNF-α.

Etanercept was utilized by Avunduk et al. (2004) [86] in the endotoxin-induced model. Etanercept substantially decreases leukocyte rolling and adhesion in the endotoxin-induced model. However, the routine clinical use of etanercept revealed a trend for the drug to induce the uveitic process; consequently, this treatment is no longer administered to patients with uveitis [87]. 

Table 1 summarizes several examples of mAbs used in relevant ocular diseases.

## 4. Conclusions

The emerging development and use of monoclonal antibodies (mAbs) has heralded a new era in the treatment of eye diseases, offering unprecedented specificity and a diverse array of mechanisms against a range of diseases. mAbs have significantly enhanced the therapeutic armamentarium against a broad spectrum of malignancies. The mechanisms of action, including inhibition of signaling pathways, antibody-dependent cellular cytotoxicity, complement-dependent cytotoxicity, and antibody-dependent cellular phagocytosis, underscore the multifaceted nature of mAb therapy. As research continues to uncover new targets and refine existing treatments, the future of ocular disease treatments is poised to benefit even more from the precision and efficacy of mAbs. Despite the advantages of treating ocular diseases with currently available mAbs, there are still gaps to be studied in order to further improve visual gains and the durability of treatment response, as well as to reduce costs.

## Figures and Tables

**Table 1:** Summary of relevant examples of monoclonal antibodies for the treatment of the main ocular diseases.

Ocular Disease	Monoclonal Antibody	Target	References
Age-related macular degeneration	Ranibizumab	VEGF-A	[88]
	Brolucizumab	VEGF-A	[89,90]
	Bevacizumab	VEGF	[91]
	Aflibercept	VEGF	[92,93]
	Conbercept	VEGF	[94]
	Faricimab	VEGF; Ang-2	[92,95]
Uveitis	Daclizumab	IL2	[96]
	Infliximab	TNF	[97]
	Etanercept	TNF	[97]
	Golimumab	TNF	[96]
	Secukinumab	IL-17A	[96]
Neovascular glaucoma	Bevacizumab	VEGF	[98]
	Aflibercept	VEGF	[98]
Diabetic macular edema	Aflibercept	VEGF	[99]
	Bevacizumab	VEGF	[99]
	Brolucizumab	VEGF-A	[90]
	Faricimab	VEGF; Ang-2	[95]
Macular edema due to retinal vein occlusion	Aflibercept	VEGF	[98]
	Faricimab	VEGF; Ang-2	[100]
	Bevacizumab	VEGF	[98,101]
Myopic choroidal neovascularization	Aflibercept	VEGF	[102,103]
	Bevacizumab	VEGF	[104,105]
	Ranibizumab	VEGF-A	[103,106]

## References

[1] Breedveld F. (2000). Therapeutic monoclonal antibodies. Lancet.

[2] Bayer V. (2019). An overview of monoclonal antibodies. Semin. Oncol. Nurs..

[3] Ecker D.M., Jones S.D., Levine H.L. (2015). The therapeutic monoclonal antibody market. mAbs.

[4] Rodríguez-Nava C., Ortuño-Pineda C., Illades-Aguiar B., Flores-Alfaro E., Leyva-Vázquez M.A., Parra-Rojas I., del Moral-Hernández O., Vences-Velázquez A., Cortés-Sarabia K., Alarcón-Romero L.d.C. (2023). Mechanisms of action and limitations of monoclonal antibodies and single chain fragment variable (scFv) in the treatment of cancer. Biomedicines.

[5] McMaster P.D., Hudack S.S. (1935). The formation of agglutinins with lymph nodes. J. Exp. Med..

[6] Singh S., Tank N.K., Dwiwedi P., Charan J., Kaur R., Sidhu P., Chugh V.K. (2018). Monoclonal antibodies: A review. Curr. Clin. Pharmacol..

[7] Rodrigues E.B., Farah M.E., Maia M., Penha F.M., Regatieri C., Melo G.B., Pinheiro M.M., Zanetti C.R. (2009). Therapeutic monoclonal antibodies in ophthalmology. Prog. Retin. Eye Res..

[8] Shibuya M. (2011). Vascular Endothelial Growth Factor (VEGF) and Its Receptor (VEGFR) Signaling in Angiogenesis: A Crucial Target for Anti- and Pro-Angiogenic Therapies. Genes Cancer.

[9] Penn J.S., Madan A., Caldwell R.B., Bartoli M., Caldwell R.W., Hartnett M.E. (2008). Vascular endothelial growth factor in eye disease. Prog. Retin. Eye Res..

[10] Lad E.M., Finger R.P., Guymer R. (2023). Biomarkers for the Progression of Intermediate Age-Related Macular Degeneration. Ophthalmol. Ther..

[11] Thomas C.J., Mirza R.G., Gill M.K. (2021). Age-Related Macular Degeneration. Med. Clin. N. Am..

[12] Stahl A. (2020). The Diagnosis and Treatment of Age-Related Macular Degeneration. Dtsch. Arztebl. Int..

[13] Stone E.M. (2007). Macular degeneration. Annu. Rev. Med..

[14] Kikushima W., Sakurada Y., Fukuda Y., Matsubara M., Kotoda Y., Sugiyama A., Kashiwagi K. (2023). A Treat-and-Extend Regimen of Intravitreal Brolucizumab for Exudative Age-Related Macular Degeneration Refractory to Aflibercept: A 12-Month Result. Pharmaceuticals.

[15] Coleman H.R., Chan C.-C., Ferris F.L., Chew E.Y. (2008). Age-related macular degeneration. Lancet.

[16] Lim L.S., Mitchell P., Seddon J.M., Holz F.G., Wong T.Y. (2012). Age-related macular degeneration. Lancet.

[17] Domalpally A., Xing B., Pak J.W., Agrón E., Ferris F.L., Clemons T.E., Chew E.Y. (2023). Extramacular Drusen and Progression of Age-Related Macular Degeneration: Age Related Eye Disease Study 2 Report 30. Ophthalmol. Retin..

[18] Flores R., Carneiro Â., Vieira M., Tenreiro S., Seabra M.C. (2021). Age-Related Macular Degeneration: Pathophysiology, Management, and Future Perspectives. Ophthalmologica.

[19] Leitch I.M., Gerometta M., Eichenbaum D., Finger R.P., Steinle N.C., Baldwin M.E. (2024). Vascular Endothelial Growth Factor C and D Signaling Pathways as Potential Targets for the Treatment of Neovascular Age-Related Macular Degeneration: A Narrative Review. Ophthalmol. Ther..

[20] Rosenfeld P.J., Rich R.M., Lalwani G.A. (2006). Ranibizumab: Phase III clinical trial results. Ophthalmol. Clin. N. Am..

[21] Wang W., Lo A.C. (2018). Diabetic retinopathy: Pathophysiology and treatments. Int. J. Mol. Sci..

[22] Fong D.S., Aiello L.P., Ferris F.L., Klein R. (2004). Diabetic retinopathy. Diabetes Care.

[23] Porta M., Bandello F. (2002). Diabetic retinopathy: A clinical update. Diabetologia.

[24] Ferris F.L., Davis M.D., Aiello L.M. (1999). Treatment of diabetic retinopathy. N. Engl. J. Med..

[25] Akinsoji E., Goldhardt R., Galor A. (2018). A Glimpse into Uveitis in the Aging Eye: Pathophysiology, Clinical Presentation and Treatment Considerations. Drugs Aging.

[26] Cuartero-Martínez A., García-Otero X., Codesido J., Gómez-Lado N., Mateos J., Bravo S.B., Rodríguez-Fernández C.A., González-Barcia M., Aguiar P., Ortega-Hortas M. (2024). Preclinical characterization of endotoxin-induced uveitis models using OCT, PET/CT and proteomics. Int. J. Pharm..

[27] Muñoz-Fernández S., Martín-Mola E. (2006). Uveitis. Best Pract. Res. Clin. Rheumatol..

[28] Chang J.H.-M., Wakefield D. (2002). Uveitis: A global perspective. Ocul. Immunol. Inflamm..

[29] de Vos A.F., van Haren M.A., Verhagen C., Hoekzema R., Kijlstra A. (1994). Kinetics of intraocular tumor necrosis factor and interleukin-6 in endotoxin-induced uveitis in the rat. Investig. Ophthalmol. Vis. Sci..

[30] Santos Lacomba M., Marcos Martín C., Gallardo Galera J.M., Gómez Vidal M.A., Collantes Estévez E., Ramírez Chamond R., Omar M. (2001). Aqueous humor and serum tumor necrosis factor-alpha in clinical uveitis. Ophthalmic Res..

[31] Santos M.F.C., Mirada G.S., do Couto J.O., de Oliveira Costa G., Rangel Rosa A.C., Gambeta Borges C.H., Crevelin E.J., de Araujo L.S., Bastos J.K., Veneziani R.C.S. (2024). A validated ultra-performance liquid chromatography with tandem mass spectrometry method for the quantification of Brazilian green propolis main compounds. Nat. Prod. Res..

[32] Leung E.H., Fan J., Flynn H.W., Albini T.A. (2022). Ocular and Systemic Complications of COVID-19: Impact on Patients and Healthcare. Clin. Ophthalmol..

[33] Siemieniuk R.A., Bartoszko J.J., Díaz Martinez J.P., Kum E., Qasim A., Zeraatkar D., Izcovich A., Mangala S., Ge L., Han M.A. (2021). Antibody and cellular therapies for treatment of COVID-19: A living systematic review and network meta-analysis. BMJ.

[34] Braghiroli M.I., Sabbaga J., Hoff P.M. (2012). Bevacizumab: Overview of the literature. Expert Rev. Anticancer Ther..

[35] Kazazi-Hyseni F., Beijnen J.H., Schellens J.H. (2010). Bevacizumab. Oncologist.

[36] Singh R.P., Avery R.L., Barakat M.R., Kim J.E., Kiss S. (2024). Evidence-Based Use of Bevacizumab in the Management of Neovascular Age-Related Macular Degeneration. Ophthalmic Surg. Lasers Imaging Retin..

[37] Estarreja J., Mendes P., Silva C., Camacho P., Mateus V. (2024). Off-Label Use of Bevacizumab in Patients Diagnosed with Age-Related Macular Degeneration: A Systematic Review and Meta-Analysis. Pharmaceuticals.

[38] Gomez-Lumbreras A., Ghule P., Panchal R., Giannouchos T., Lockhart C.M., Brixner D. (2023). Real-world evidence in the use of Bevacizumab in age-related macular degeneration (ArMD): A scoping review. Int. Ophthalmol..

[39] Gumus G., Berhuni M., Ozturkmen C. (2022). The short-term effects of intravitreal bevacizumab injection on intraocular pressure, cornea, iridocorneal angle, and anterior chamber. Ther. Adv. Ophthalmol..

[40] Blick S.K., Keating G.M., Wagstaff A.J. (2007). Ranibizumab. Drugs.

[41] Triantafylla M., Massa H.F., Dardabounis D., Gatzioufas Z., Kozobolis V., Ioannakis K., Perente I., Panos G.D. (2014). Ranibizumab for the treatment of degenerative ocular conditions. Clin. Ophthalmol..

[42] Pershing S., Talwar N., Armenti S.T., Grubbs J., Rosenthal J.M., Dedania V.S., Stein J.D. (2019). Use of Bevacizumab and Ranibizumab for Wet Age-Related Macular Degeneration: Influence of CATT Results and Introduction of Aflibercept. Am. J. Ophthalmol..

[43] Parravano M., Costanzo E., Scondotto G., Trifirò G., Virgili G. (2021). Anti-VEGF and Other Novel Therapies for Neovascular Age-Related Macular Degeneration: An Update. BioDrugs.

[44] Scheinfeld N. (2005). Adalimumab: A review of side effects. Expert Opin. Drug Saf..

[45] Rudwaleit M., Rødevand E., Holck P., Vanhoof J., Kron M., Kary S., Kupper H. (2009). Adalimumab effectively reduces the rate of anterior uveitis flares in patients with active ankylosing spondylitis: Results of a prospective open-label study. Ann. Rheum. Dis..

[46] Takase K., Ohno S., Ideguchi H., Uchio E., Takeno M., Ishigatsubo Y. (2011). Successful switching to adalimumab in an infliximab-allergic patient with severe Behçet disease-related uveitis. Rheumatol. Int..

[47] Diaz-Llopis M., García-Delpech S., Salom D., Udaondo P., Hernández-Garfella M., Bosch-Morell F., Quijada A., Romero F.J. (2008). Adalimumab therapy for refractory uveitis: A pilot study. J. Ocul. Pharmacol. Ther..

[48] Winterfield L.S., Menter A. (2004). Infliximab. Dermatol. Ther..

[49] Miraldi Utz V., Bulas S., Lopper S., Fenchel M., Sa T., Mehta M., Ash D., Lovell D.J., Kaufman A.H. (2019). Effectiveness of long-term infliximab use and impact of treatment adherence on disease control in refractory, non-infectious pediatric uveitis. Pediatr. Rheumatol..

[50] Cordero-Coma M., Sobrin L. (2015). Anti-tumor necrosis factor-α therapy in uveitis. Surv. Ophthalmol..

[51] Deitch I., Amer R., Tomkins-Netzer O., Habot-Wilner Z., Friling R., Neumann R., Kramer M. (2018). The effect of anti-tumor necrosis factor alpha agents on the outcome in pediatric uveitis of diverse etiologies. Graefe’s Arch. Clin. Exp. Ophthalmol..

[52] Maleki A., Sahawneh H.F., Ma L., Meese H., He Y., Foster C.S. (2017). Infliximab therapy with noninfectious intermediate uveitis resistant to conventional immunomodulatory therapy. Retina.

[53] Cunningham E.T. (2010). Exogenous factors influencing endogenous inflammation: What can patients do to improve control of their own uveitis?. Br. J. Ophthalmol..

[54] Dolz-Marco R., Gallego-Pinazo R., Díaz-Llopis M., Cunningham E.T., Arévalo J.F. (2015). Noninfectious uveitis: Strategies to optimize treatment compliance and adherence. Clin. Ophthalmol..

[55] Mushtaq B., Saeed T., Situnayake R.D., Murray P.I. (2007). Adalimumab for sight-threatening uveitis in Behçet’s disease. Eye.

[56] Llorenç V., Mesquida M., Sainz de la Maza M., Blanco R., Calvo V., Maíz O., Blanco A., de Dios-Jiménez de Aberásturi J.R., Adán A. (2016). Certolizumab Pegol, a New Anti-TNF-α in the Armamentarium against Ocular Inflammation. Ocul. Immunol. Inflamm..

[57] Lee J.U., Shin W., Son J.Y., Yoo K.Y., Heo Y.S. (2017). Molecular Basis for the Neutralization of Tumor Necrosis Factor α by Certolizumab Pegol in the Treatment of Inflammatory Autoimmune Diseases. Int. J. Mol. Sci..

[58] Frías E.D., González J.F.D. (2011). Certolizumab pegol. Reumatol. Clin..

[59] Moisseiev E., Shulman S. (2014). Certolizumab-induced uveitis: A case report and review of the literature. Case Rep. Ophthalmol..

[60] Sharon Y., Chu D.S. (2020). Certolizumab pegol—Tumor necrosis factor inhibitor for refractory uveitis. Am. J. Ophthalmol. Case Rep..

[61] Benschop R.J., Tuttle J.L., Zhang L., Poorbaugh J., Kallewaard N.L., Vaillancourt P., Crisp M., Trinh T.N.V., Freitas J.J., Beasley S. (2022). The anti-SARS-CoV-2 monoclonal antibody bamlanivimab minimally affects the endogenous immune response to COVID-19 vaccination. Sci. Transl. Med..

[62] Gottlieb R.L., Nirula A., Chen P., Boscia J., Heller B., Morris J., Huhn G., Cardona J., Mocherla B., Stosor V. (2021). Effect of Bamlanivimab as Monotherapy or in Combination with Etesevimab on Viral Load in Patients With Mild to Moderate COVID-19: A Randomized Clinical Trial. JAMA.

[63] Deeks E.D. (2021). Casirivimab/imdevimab: First approval. Drugs.

[64] Vellas C., Del Bello A., Gaube G., Tremeaux P., Jeanne N., Ranger N., Martin-Blondel G., Delobel P., Kamar N., Izopet J. (2022). Impact of Casirivimab-Imdevimab on Severe Acute Respiratory Syndrome Coronavirus 2 Delta Variant Nasopharyngeal Virus Load and Spike Quasispecies. Open Forum Infect. Dis..

[65] Hansen J., Baum A., Pascal K.E., Russo V., Giordano S., Wloga E., Fulton B.O., Yan Y., Koon K., Patel K. (2020). Studies in humanized mice and convalescent humans yield a SARS-CoV-2 antibody cocktail. Science.

[66] Baumal C.R., Bodaghi B., Singer M., Tanzer D.J., Seres A., Joshi M.R., Feltgen N., Gale R. (2021). Expert Opinion on Management of Intraocular Inflammation, Retinal Vasculitis, and Vascular Occlusion after Brolucizumab Treatment. Ophthalmol. Retin..

[67] Dugel P.U., Singh R.P., Koh A., Ogura Y., Weissgerber G., Gedif K., Jaffe G.J., Tadayoni R., Schmidt-Erfurth U., Holz F.G. (2021). HAWK and HARRIER: Ninety-Six-Week Outcomes from the Phase 3 Trials of Brolucizumab for Neovascular Age-Related Macular Degeneration. Ophthalmology.

[68] Nguyen H.V., Li A.S., Silva A.R., Leng T. (2022). Ocular adverse events following intravitreal brolucizumab for neovascular age-related macular degeneration at a single tertiary care center. Eur. J. Ophthalmol..

[69] Abu Serhan H., Taha M.J.J., Abuawwad M.T., Abdelaal A., Irshaidat S., Abu Serhan L., Abu Salim Q.F., Awamleh N., Abdelazeem B., Elnahry A.G. (2024). Safety and Efficacy of Brolucizumab in the Treatment of Diabetic Macular Edema and Diabetic Retinopathy: A Systematic Review and Meta-Analysis. Semin. Ophthalmol..

[70] Brown D.M., Emanuelli A., Bandello F., Barranco J.J.E., Figueira J., Souied E., Wolf S., Gupta V., Ngah N.F., Liew G. (2022). KESTREL and KITE: 52-Week Results From Two Phase III Pivotal Trials of Brolucizumab for Diabetic Macular Edema. Am. J. Ophthalmol..

[71] Hirano T., Kumazaki A., Tomihara R., Ito S., Hoshiyama K., Murata T. (2023). Evaluating initial responses to brolucizumab in patients undergoing conventional anti-VEGF therapy for diabetic macular edema: A retrospective, single-center, observational study. Sci. Rep..

[72] Kuo B.L., Singh R.P. (2022). Brolucizumab for the treatment of diabetic macular edema. Curr. Opin. Ophthalmol..

[73] Singh R.P., Barakat M.R., Ip M.S., Wykoff C.C., Eichenbaum D.A., Joshi S., Warrow D., Sheth V.S., Stefanickova J., Kim Y.S. (2023). Efficacy and Safety of Brolucizumab for Diabetic Macular Edema: The KINGFISHER Randomized Clinical Trial. JAMA Ophthalmol..

[74] Ferro Desideri L., Traverso C.E., Nicolò M., Munk M.R. (2023). Faricimab for the treatment of diabetic macular edema and neovascular age-related macular degeneration. Pharmaceutics.

[75] Shimura M., Kitano S., Ogata N., Mitamura Y., Oh H., Ochi H., Ohsawa S., Hirakata A., Bolz M., Findl O. (2023). Efficacy, durability, and safety of faricimab with extended dosing up to every 16 weeks in Japanese patients with diabetic macular edema: 1-year results from the Japan subgroup of the phase 3 YOSEMITE trial. Jpn. J. Ophthalmol..

[76] Agostini H., Abreu F., Baumal C.R., Chang D.S., Csaky K.G., Demetriades A.M., Kodjikian L., Lim J.I., Margaron P., Monés J.M. (2024). Faricimab for neovascular age-related macular degeneration and diabetic macular edema: From preclinical studies to phase 3 outcomes. Graefe’s Arch. Clin. Exp. Ophthalmol..

[77] Takahashi H., Inoda S., Takahashi H., Takahashi R., Hashimoto Y., Yoshida H., Kawashima H., Yanagi Y. (2024). One-year visual and anatomical outcomes of intravitreal faricimab injection for neovascular age-related macular degeneration after prior brolucizumab treatment. Sci. Rep..

[78] Khanani A.M., Guymer R.H., Basu K., Boston H., Heier J.S., Korobelnik J.F., Kotecha A., Lin H., Silverman D., Swaminathan B. (2021). TENAYA and LUCERNE: Rationale and Design for the Phase 3 Clinical Trials of Faricimab for Neovascular Age-Related Macular Degeneration. Ophthalmol. Sci..

[79] Heier J.S., Khanani A.M., Quezada Ruiz C., Basu K., Ferrone P.J., Brittain C., Figueroa M.S., Lin H., Holz F.G., Patel V. (2022). Efficacy, durability, and safety of intravitreal faricimab up to every 16 weeks for neovascular age-related macular degeneration (TENAYA and LUCERNE): Two randomised, double-masked, phase 3, non-inferiority trials. Lancet.

[80] Śpiewak D., Drzyzga Ł., Dorecka M., Wyględowska-Promieńska D. (2024). Summary of the Therapeutic Options for Patients with Dry and Neovascular AMD. J. Clin. Med..

[81] DeCroos F.C., Reed D., Adam M.K., Salz D., Gupta O.P., Ho A.C., Regillo C.D. (2017). Treat-and-Extend Therapy Using Aflibercept for Neovascular Age-related Macular Degeneration: A Prospective Clinical Trial. Am. J. Ophthalmol..

[82] Lanzetta P., Korobelnik J.F., Heier J.S., Leal S., Holz F.G., Clark W.L., Eichenbaum D., Iida T., Xiaodong S., Berliner A.J. (2024). Intravitreal aflibercept 8 mg in neovascular age-related macular degeneration (PULSAR): 48-week results from a randomised, double-masked, non-inferiority, phase 3 trial. Lancet.

[83] Adams B.S., Sorhaitz W., Stringham J. (2023). Aflibercept. StatPearls.

[84] de Oliveira Dias J.R., Badaró E., Novais E.A., Colicchio D., Chiarantin G.M., Matioli M.M., Verna C., Penha F.M., Barros N.M., Meyer C.H. (2014). Preclinical investigations of intravitreal ziv-aflibercept. Ophthalmic Surg. Lasers Imaging Retin..

[85] Iyer P.G., Albini T.A. (2021). Drug-related adverse effects of antivascular endothelial growth factor agents. Curr. Opin. Ophthalmol..

[86] Avunduk M.C., Avunduk A.M., Oztekin E., Baltaci A.K., Ozyazgan Y., Mogolkoc R. (2004). Etanercept treatment in the endotoxin-induced uveitis of rats. Exp. Eye Res..

[87] Lim L.L., Fraunfelder F.W., Rosenbaum J.T. (2007). Do tumor necrosis factor inhibitors cause uveitis? A registry-based study. Arthritis Rheum..

[88] García-Quintanilla L., Almuiña-Varela P., Maroñas O., Gil-Rodriguez A., Rodríguez-Cid M.J., Gil-Martinez M., Abraldes M.J., Gómez-Ulla de Irazazabal F., González-Barcia M., Mondelo-Garcia C. (2023). Influence of Genetic Polymorphisms on the Short-Term Response to Ranibizumab in Patients With Neovascular Age-Related Macular Degeneration. Investig. Ophthalmol. Vis. Sci..

[89] Ferro Desideri L., Traverso C.E., Nicolò M. (2021). Brolucizumab: A novel anti-VEGF humanized single-chain antibody fragment for treating w-AMD. Expert Opin. Biol. Ther..

[90] Markham A. (2019). Brolucizumab: First Approval. Drugs.

[91] Shepard H.M., Phillips G.L., Thanos C.D., Feldmann M. (2017). Developments in therapy with monoclonal antibodies and related proteins. Clin. Med..

[92] Tamiya R., Hata M., Tanaka A., Tsuchikawa M., Ueda-Arakawa N., Tamura H., Miyata M., Takahashi A., Kido A., Muraoka Y. (2023). Therapeutic effects of faricimab on aflibercept-refractory age-related macular degeneration. Sci. Rep..

[93] Jaggi D., Nagamany T., Ebneter A., Munk M., Wolf S., Zinkernagel M. (2022). Aflibercept for age-related macular degeneration: 4-year outcomes of a ‘treat-and-extend’ regimen with exit-strategy. Br. J. Ophthalmol..

[94] Cheng S., Zhang S., Huang M., Liu Y., Zou X., Chen X., Zhang Z. (2024). Treatment of neovascular age-related macular degeneration with anti-vascular endothelial growth factor drugs: Progress from mechanisms to clinical applications. Front. Med..

[95] Quah N.Q.X., Javed K., Arbi L., Hanumunthadu D. (2024). Real-World Outcomes of Faricimab Treatment for Neovascular Age-Related Macular Degeneration and Diabetic Macular Edema. Clin. Ophthalmol..

[96] Schwartzman S. (2016). Advancements in the management of uveitis. Best Pract. Res. Clin. Rheumatol..

[97] Trivedi A., Katelaris C. (2019). The use of biologic agents in the management of uveitis. Intern. Med. J..

[98] Scott I.U., VanVeldhuisen P.C., Ip M.S., Blodi B.A., Oden N.L., Awh C.C., Kunimoto D.Y., Marcus D.M., Wroblewski J.J., King J. (2017). Effect of Bevacizumab vs Aflibercept on Visual Acuity Among Patients with Macular Edema Due to Central Retinal Vein Occlusion: The SCORE2 Randomized Clinical Trial. JAMA.

[99] Virgili G., Parravano M., Evans J.R., Gordon I., Lucenteforte E. (2017). Anti-vascular endothelial growth factor for diabetic macular oedema: A network meta-analysis. Cochrane Database Syst. Rev..

[100] Shirley M. (2022). Faricimab: First Approval. Drugs.

[101] Bressler N.M. (2017). Treatment of Macular Edema Due to Central Retinal Vein Occlusion: Another Score for Repackaged Bevacizumab. JAMA.

[102] Pakzad-Vaezi K., Mehta H., Mammo Z., Tufail A. (2016). Vascular endothelial growth factor inhibitor use and treatment approach for choroidal neovascularization secondary to pathologic myopia. Expert Opin. Biol. Ther..

[103] Sayanagi K., Uematsu S., Hara C., Wakabayashi T., Fukushima Y., Sato S., Ikuno Y., Nishida K. (2019). Effect of intravitreal injection of aflibercept or ranibizumab on chorioretinal atrophy in myopic choroidal neovascularization. Graefe’s Arch. Clin. Exp. Ophthalmol..

[104] Ruiz-Moreno J.M., Montero J.A., Amat-Peral P. (2011). Myopic choroidal neovascularization treated by intravitreal bevacizumab: Comparison of two different initial doses. Graefe’s Arch. Clin. Exp. Ophthalmol..

[105] Toto L., Di Antonio L., Costantino O., Mastropasqua R. (2021). Anti-VEGF Therapy in Myopic CNV. Curr. Drug Targets.

[106] Ng D.S.C., Fung N.S.K., Yip F.L.T., Lai T.Y.Y. (2020). Ranibizumab for myopic choroidal neovascularization. Expert Opin. Biol. Ther..

